# Optimising Primary thErapy in pRimAry biliary cholangitis (OPERA): protocol for a randomised, double-blind, placebo-controlled trial of enhanced primary therapy with obeticholic acid

**DOI:** 10.1136/bmjopen-2025-113812

**Published:** 2026-03-11

**Authors:** Sarah Dunn, Lysbeth Evans, Ciara Kennedy, Rebecca Wafer, Sam Moody, Faye Wolstenhulme, Emma Burton, Ann Breeze Konkoth, Holly Fisher, Thomas Chadwick, Annie Banham, Fiona Hale, Mo Christie, Stephen T Barclay, Jessica Dyson, Rachel Smith, Guruprasad Aithal, Emma Culver, Richard Aspinall, Doug Thorburn, Michael Heneghan, Helen C Hancock, Palak Trivedi, George Mells, James Wason, David E J Jones

**Affiliations:** 1 Newcastle Clinical Trials Unit, Newcastle University, Newcastle upon Tyne, UK; 2 Newcastle University, Newcastle upon Tyne, UK; 3 Population Health Sciences Institute, Newcastle University Faculty of Medical Sciences, Newcastle upon Tyne, UK; 4 Newcastle University Institute for Health and Society, Newcastle upon Tyne, UK; 5 PPIE, Newcastle upon tyne, UK; 6 PBC Foundation, Edinburgh, UK; 7 Glasgow Royal Infirmary, Glasgow, UK; 8 Newcastle Upon Tyne Hospitals NHS Foundation Trust, Newcastle upon Tyne, UK; 9 Cambridge University Hospitals NHS Foundation Trust, Cambridge, UK; 10 Wofson Digestive Disease Centre, Queens Medical Centre University Hospital, Nottingham, UK; 11 Oxford University Hospitals NHS Foundation Trust, Oxford, UK; 12 Portsmouth Hospitals University NHS Trust, Portsmouth, UK; 13 Department of Hepatology, Royal Free London NHS Trust, London, UK; 14 Institute of Liver Studies, King’s College London, London, UK; 15 University Hospitals Birmingham NHS Foundation Trust, Birmingham, UK; 16 Population Health Sciences Institute, Newcastle University, Newcastle upon Tyne, UK; 17 MRC Biostatistics Unit, University of Cambridge, Cambridge, UK; 18 NIHR Newcastle Biomedical Research Centre and Institute of Cellular Medicine, Newcastle University, Newcastle upon Tyne, UK

**Keywords:** Hepatology, Clinical Trial, Disease Management, Clinical Protocols, Hepatobiliary disease, Randomized Controlled Trial

## Abstract

**Introduction:**

Primary biliary cholangitis (PBC) is a rare chronic cholestatic disease that despite current therapy has significant ongoing unmet needs, including risks of cirrhosis and life-impairing symptoms. The current treatment approach is a step-up model, wherein first-line therapy, ursodeoxycholic acid (UDCA), is given for a minimum of 12 months before the addition of second-line therapy is considered for non-responding patients. This ‘waiting to fail’ approach, focused on the needs of low-risk patients, allows, we postulate, a key process of biliary epithelial cell (BEC) senescence to become established, driving accelerated bile duct loss and aggressive disease. Preclinical mouse modelling has shown that early use of the farnesoid X receptor agonist obeticholic acid (OCA), currently only used as second-line therapy following UDCA failure, reverses BEC senescence, changing the clinical course of disease. Here, we describe the design of the Optimising Primary thErapy in pRimAry biliary cholangitis (OPERA) trial. The aim of OPERA is to explore a new paradigm for disease-modifying treatment of PBC: risk-informed early treatment stratification, with patients at increased risk offered UDCA and OCA combination with the goal of complete biochemical remission.

**Methods and analysis:**

OPERA is a multicentre, randomised, double-blind, placebo-controlled trial of OCA in combination with UDCA, as first-line treatment for high-risk PBC. This is a multicentre trial in England, which will be undertaken in specialist clinics in secondary/tertiary referral centres (or as per local set up). These centres will be specialists in the area of PBC management and will manage patients from across their local region. OPERA will recruit and randomise 106 adults, within 6 months of PBC diagnosis, who are at an enhanced risk of non-response to standard first-line therapy, between either: (1) UDCA and OCA or (2) UDCA and matched placebo in a 1:1 ratio. The primary efficacy outcome measure is the percentage of participants showing normalisation of serum alkaline phosphatase and total bilirubin values at 26 weeks (disease remission).

**Ethics and dissemination:**

Favourable ethical opinion was received from London – Riverside Research Ethics Committee (reference: 22/LO/0878). Potential participants will be fully informed of their rights and the benefits and harms of the trial by the research team before giving informed consent to participate in the trial. Results will be disseminated in peer-reviewed publications, at national and international conferences, in peer-reviewed journals and to participants and the public (using lay language).

**Trial registration number:**

ISRCTN17176388.

**STRENGTHS AND LIMITATIONS OF THIS STUDY**

- This protocol presents a randomised controlled trial designed to evaluate the effect of obeticholic acid (OCA) as a first-line therapy for primary biliary cholangitis patients, challenging the potentially flawed ‘step up’ therapy model that potentially leaves the highest risk patients untreated for a significant period of time, assessing the impact of a more contemporary top-down, disease modification approach.
- The trial uses biochemical remission (normalisation in serum alkaline phosphatase values) as the efficacy outcome measure, as opposed to the conventional, but flawed, biochemical response indices, which tolerate ongoing disease activity.
- A biopsy sub-study will allow, for the first time, the mechanism of OCA on bile duct injury and, in particular, the role played by cellular senescence to be addressed.
- The trial uses a novel statistical method to reduce the sample size required without sacrificing statistical power.
- The trial relies on a biochemical surrogate marker of outcome, as it is not designed or powered to explore true clinical outcomes; additionally, it only explores one of the potential therapies applicable in this setting.

## Introduction

 Primary biliary cholangitis (PBC) is a rare chronic cholestatic liver disease with significant unmet needs despite current therapy. PBC prevalence is 40/100 000 in the UK with an estimated incidence of 1–2/100 000 per year.^1^ Clinical impact in PBC comes from the risk of progression to cirrhosis with its associated complications and, alongside, difficult-to-treat symptoms; specifically pruritus, fatigue and cognitive dysfunction, that can occur at any point in the disease course, and which result in significant impact on health utility.^2^,^9^ Current standard care is a step-up model, wherein first-line ursodeoxycholic acid (UDCA) is given for a minimum of 12 months before more effective ‘add-on’ therapy is considered for non-responding or under-responding patients.

At a population level, UDCA improves serum liver biochemical markers, delays liver transplantation and increases life expectancy.^210^,^13^ However, approximately 40% of UDCA-treated patients still go on to develop cirrhosis (40% in a multicentre study of >1000 patients over 10 years^14^; 46% of patients in contemporary USA ‘real world data’ report of 15 875 patients,^15^ 60% display persistently abnormal liver blood tests despite UDCA (59% in a UK-PBC nested cohort of 400 patients)^16^ and >80% continue to experience symptoms, especially fatigue and cognitive symptoms that are seemingly not modified by any current intervention.^2 3 9 17^

Current treatment paradigms grant access to the licensed second-line therapy obeticholic acid (OCA; a first in class farnesoid X receptor agonist) given in addition to UDCA, only after patients are deemed to be ‘non-responders’ to UDCA. This ‘step-up’ approach means that the most effective agent is only used after first-line therapy has been tested (and failed) for at least a year.

In PBC, UDCA treatment failure does not occur at random; studies from UK-PBC have clearly shown that UDCA treatment failure can be accurately predicted by pretreatment clinical parameters including the patient’s age and serum biochemistry.^10^ These parameters can be used to produce an accurate predictive UDCA Response Score (URS) that has been validated in Italian and Japanese as well as UK populations.^18^

The impact of waiting to institute more effective therapy in UDCA non-responders goes beyond delaying deriving clinical benefit. The lost time may actually change the trajectory of the disease by allowing key disease processes to become embedded. Critically, UK-PBC studies have demonstrated that therapy delay in PBC leads to a significantly reduced likelihood that the therapy will work when eventually used.^10^ The importance of biliary epithelial cell (BEC) senescence in disease pathogenesis, its contribution to the high-risk phenotype (including progression to irreversible bile duct loss), and the fact that it begins early in the disease process in high-risk patients (and is progressive thereafter) affords a potential explanation as to why treatment delay results in reduced effectiveness. This emphasises the need for earlier, better and more efficient treatment models—a concept that underpins the Optimising Primary thErapy in pRimAry (OPERA) trial concept.

In the OPERA trial, we will evaluate a novel treatment strategy in newly presenting PBC patients; we will use the validated URS to identify, at the point of disease diagnosis, patients who are at reduced likelihood of achieving biochemical remission in response to UDCA monotherapy, and will implement a risk-determined stratified approach whereby the licensed second-line therapy (OCA) is used from the outset in combination with existing first-line therapy.^10^ This strategy will allow us to optimise treatment delivery in the higher risk group early on, without over-treating the lower risk patients. We will also adopt an emerging, evidence-based primary outcome of complete disease control (normalisation of liver biochemistry).

This trial will adopt a dosing regimen optimal for reaching effective doses of OCA, while avoiding pruritus in a trial design setting where liver function test levels are not appropriate for use in assessing efficacy and guiding dose escalation.

As noted in, Clinical effectiveness of Obeticholic Acid (section 4.4) of the NICE guideline for OCA in PBC^19^ and based on the findings of the POISE Trial,^20^ clinical effectiveness of OCA would be better measured at 10 mg. The OPERA trial will close this gap in the literature by measuring an observable disease modifying effect within the trial time frame of 26 weeks. 26 weeks is long enough to observe a disease modifying effect and short enough to allow discontinuation of therapy.^21 22^ If a relapse is experienced by the participant after 26 weeks, they will be assessed for suitability to introduce OCA at 1 year post-UDCA start, according to current National Health Service (NHS) standard of care.^23^

In parallel, we will validate and explore the clinical utility of a mechanistic composite biomarker linked to BEC senescence, based around circulating levels of the chemokines CCL20 and CXCL11.^24^ These parameters will be used to predict and quantify the response to second-line therapy instituted early, comparing it to clinical outcomes, and to the gold standard of liver biopsy assessment of BEC senescence. This marker, developed in UK-PBC pilot studies, has a positive predictive value of 94%, a negative predictive value of 78%, and an area under the receiver operator curve of 0.91 for the identification of UDCA non-responding patients stratified using the current clinical criteria.^24^

## Methods and analysis

### Trial design and setting

OPERA is a double-blinded, superiority, randomised, placebo-controlled, multi-centre clinical trial of an investigational medicinal product (CTIMP). This is a multicentre trial in England, which will be undertaken in specialist clinics in secondary/tertiary referral centres (or as per local set up). These centres will be specialists in the area of PBC management and will manage patients from across their local region. To improve trial efficiency, we will also use an innovative augmented binary analysis method^25^ to improve the precision of a trial where a dichotomous endpoint is derived from continuous data. Using this method substantially reduces the sample size needed for a power of 90%.

The trial population will be newly diagnosed, non-cirrhotic PBC patients, with less than 3 months treatment with UDCA at the time of consent and at an enhanced risk of not achieving biochemical disease remission (based on URS) with UDCA first-line therapy alone. Potentially eligible participants will be invited for a screening visit where informed consent will be received (online supplemental material). Patients will be encouraged to ask questions and will be informed of their right to withdraw from the trial. Potential participants will have sufficient time to review the trial documentation prior to this screening visit. Consent for the optional biopsy sub-study will be through an optional clause on the main trial informed consent form. The trial comprises a 26-week treatment phase in a cohort of 106 participants (OCA n=53; placebo n=53), followed by a 26-week follow-up period. All participants will continue to receive standard of care background treatment with UDCA throughout the trial.

### Objectives and outcomes

The primary and secondary objectives for the trial are described in table 1.

**Table 1:** Objectives and outcome measures

Objectives	Outcome measures
Primary	
To assess the impact of first-line obeticholic acid therapy combined with UDCA, compared with placebo combined with UDCA, in achieving biochemical remission of disease in new onset PBC patients with an enhanced disease risk.	Percentage of participants showing normalisation of serum alkaline phosphatase and total bilirubin levels at 26 weeks (visit 5)—defined as biochemical remission.
Secondary	
(a) To assess whether biochemical remission is sustained following discontinuation of experimental therapy and reversion to UDCA standard of care therapy.	(a) Percentage of participants in each arm showing sustained normalisation of serum alkaline phosphatase and total bilirubin levels at 52 weeks (visit 6).
(b) To assess the degree of biochemical improvement with obeticholic acid therapy combined with UDCA standard of care therapy compared with placebo.	(b) Magnitude of alkaline phosphatase and bilirubin reduction from baseline to 26 weeks (visit 5), assessed as a continuous variable.
(c) To assess impact of obeticholic acid therapy combined with UDCA standard of care therapy compared with placebo using conventional therapy response criteria (as used in current clinical practice).	(c) Percentage of participants attaining serum alkaline phosphatase values lower than 1.67× the upper limit of normal and a bilirubin <1× the upper limit of normal at 26 weeks (visit 5). These are termed the POISE criteria and are widely applied as outcome measures in conventional clinical trials of second-line PBC therapy. Change in liver stiffness assessed by Transient Elastography (FibroScan) from screening to week 26.
(d) To assess the safety and tolerability of obeticholic acid as first line therapy in PBC.	(d) AE and SAE Reporting up to 26 weeks (visit 5).
(e) To assess the impact of the intervention compared with placebo on symptom severity and participant quality of life.	(e) PBC-40 (change from baseline to 26 weeks (visit 5) and 52 weeks (visit 6)). (f) EQ-5D-5L (change from baseline to 26 weeks (visit 5) and 52 weeks (visit 6)). (g) Patient Health Questionnaire (26 and 52 weeks).
Experimental	
(a) To assess whether biochemical remission is sustained following discontinuation of experimental therapy and reversion to UDCA standard of care therapy in those participants who are in remission at 26 weeks.	(a) Percentage of participants in each trial group who remain in remission at 52 weeks (visit 6) as a proportion of those who showed normalisation of serum alkaline phosphatase and total bilirubin levels at primary end-point assessment at 26 weeks (visit 5).
(b) To assess changes in chemokine levels in the blood potentially associated with bile duct senescence.	(b) Serum levels of putative senescence-associated chemokines, demonstrated to be elevated in high-risk disease and UDCA non-responders in underpinning UK-PBC studies as outlined earlier, will be quantified using a bespoke multiplex assay and evaluated as dynamic risk and response markers. The combination of CCL20 and CXCL11 will be specifically explored as a baseline and early response predictive biomarker and validated against liver biopsy findings. Assessment of the capacity for ongoing elevation of chemokine biomarkers at 26 weeks to predict biochemical relapse at 52 weeks.
(c) To assess impact of intervention compared with placebo on degree of liver inflammation and bile duct senescence.	(c) Change in liver fibrosis stage on liver histology and the degree of bile duct senescence assessed using p16 and p21 immunohistochemistry in a biopsy sub-study (n approx. 15 in each group). p16 and p21 are established and well-validated cellular markers of senescence ^25^. Biopsies will be centrally read by two pathologists blinded to treatment allocation and findings correlated with serum chemokine marker values.

AE, adverse event; EQ5D5L, EuroQol - 5 Dimensions - 5 Levels; PBC, primary biliary cholangitis; POISE, Predicted Risk of End-Stage Liver Disease; SAE, serious AE; UDCA, ursodeoxycholic acid.

### Intervention

From baseline, participants will receive 5 mg once a day of OCA or placebo to be taken orally for 12 weeks. OCA or placebo will then be titrated up to 10 mg once a day from visit 4 (week 12±1 week) until visit 5 (week 26±1) according to tolerability. The participants in the OPERA trial will receive UDCA in combination with the trial IMP (OCA or matched placebo). UDCA will be prescribed in line with NHS Standard of Care (usually 13–15 mg/kg daily) and will be prescribed, dispensed and received by the participant, outside of the parameters of this trial. Intolerability to UDCA will be managed as per standard of care. Treatment with UDCA will be recorded as a concomitant medicine.

### Patient identification

Recruiting from 14 centres specialising in the area of PBC management across the UK, participants will be identified at routine clinics or by database search. Sites will use a letter of invitation and Participant Information Sheet to approach patients who are potentially eligible for the trial.

Secondary centres can also identify newly diagnosed PBC patients and, on receiving permission, refer to their local participating site.

Patient awareness will be raised through patient groups, newsletters, websites and social media. The trial will also be highlighted through local and national speaker meetings, production of posters and opportunities to discuss the trial with members of the clinical team.

### Eligibility criteria

The inclusion and exclusion criteria for the trial are described in table 2.

**Table 2:** Eligibility criteria

Inclusion criteria	Established diagnosis of PBC based on the presence of at least 2 out of the 3 key disease characteristics, specifically: Anti-mitochondrial antibody (AMA) or PBC-specific anti-nuclear antibodies (ANA) at a clinically diagnostic level. Elevated ALP (above the upper limit of normal (ULN) for the relevant laboratory). Compatible or diagnostic liver biopsy. Ongoing elevation of ALP (≥15% above ULN) at screening. Disease duration of <6 months from date of diagnosis at the time of consent. Use of UDCA for <3 months at the time of consent. Increased risk of not achieving disease remission with treatment (for the purposes of the trial defined as a pre-treatment ursodeoxycholic acid response score^10^ with a predicted risk of future non-remission of liver biochemistry (ie, ALP>ULN) with UDCA alone of >20%). For people of childbearing potential: an agreement to use at least an acceptable effective method of contraception or to practise sexual abstinence* to avoid pregnancy for the entire duration of the trial period. Willing to complete the trial assessment protocols. Ability to consent, able to comply with trial protocol and attend clinic visits. Age ≥18 years at the time of consent.
Exclusion criteria	Clinical contraindication to obeticholic acid use. Untreated, clinically significant pruritus (patients with effectively treated pruritus are eligible for inclusion). Concomitant use of fibric acid derivatives (eg, bezafibrate or fenofibrate) within 14 days prior to screening. Clinical suspicion of cirrhosis evidenced by a history of one or more of the following: ascites requiring diuretic therapy or percutaneous drainage. endoscopically confirmed varices. liver biopsy suggesting cirrhosis. platelet count <150×10^9^/L. transient elastography score >16.9 kPa within 3 months prior to or at screening. hepatocellular carcinoma confirmed by biopsy or two imaging modalities. hepatic encephalopathy. Bilirubin >twice the ULN other than in the context of Gilbert’s syndrome or another cause of unconjugated hyper-bilirubinaemia. Evidence of complete biliary obstruction. Previous exposure to obeticholic acid (either in clinical trials or in clinical practice) or other potential PBC-modifying therapy. Regular (more than 1 week per month) alcohol consumption in excess of recommended safe limits (14 units per week). Active participation in another interventional trial or exposure to another experimental drug within five half-lives. Pregnancy or planning to get pregnant within the duration of participation in the trial. Currently breastfeeding. Overlapping features of an additional liver disease, including autoimmune hepatitis (using the Paris criteria for autoimmune hepatitis overlap). Hypersensitivity to the active substance or to any of the excipients. If the participant’s treating clinician deems the patient is not suitable to participate in the trial based on other criteria apparent during screening or from medical history. Previous liver transplantation.

* Abstinence is defined as refraining from heterosexual intercourse during the entire trial period. Abstinence is acceptable only as true abstinence: when this is in line with the preferred and usual lifestyle of the patient. Periodic abstinence (eg, calendar, ovulation, symptothermal, post-ovulation methods) and withdrawal are not acceptable methods of contraception.

ALP, alkaline phosphatase; PBC, primary biliary cholangitis; UDCA, ursodeoxycholic acid.

### Consent

Potentially eligible participants will be invited for a screening visit where informed consent will be taken (online supplemental material). Screening will take place in a dedicated session held at the clinic facility in which normal care is offered, or at a research specific visit. Patients will be encouraged to ask questions about the trial and consider whether they wish to participate. The patient will be informed of their right to withdraw from the trial at any time without being subject to any resulting detriment, by revoking their informed consent.

Consent will be obtained prior to any activities undertaken as part of the screening visit (visit 1). Consent for the optional biopsy sub-study will also be obtained at this point if the participant chooses to take part.

### Randomisation

Eligible patients will be randomised to one of two treatment arms, OCA or matched placebo, in a 1:1 ratio. Treatment allocation is double blind, though emergency unblinding is available for medical or safety reasons where it is necessary for the treating clinician to know the participants’ treatment allocation.

The allocation sequence will be computer-generated, using a random permuted block design; blocks might vary in size and will not be disclosed, to ensure concealment. Patients will be randomised in a ratio of 1:1 (placebo: intervention), with stratification according to consent to the biopsy sub-study (to ensure equal allocation between arms in the sub-study).

Randomisation will be carried out using the trial database, Sealed Envelope (London, UK), a central, secure, 24-hour, web-based randomisation system with concealed allocation.

### Participant follow-up assessments

**Figure 1:**
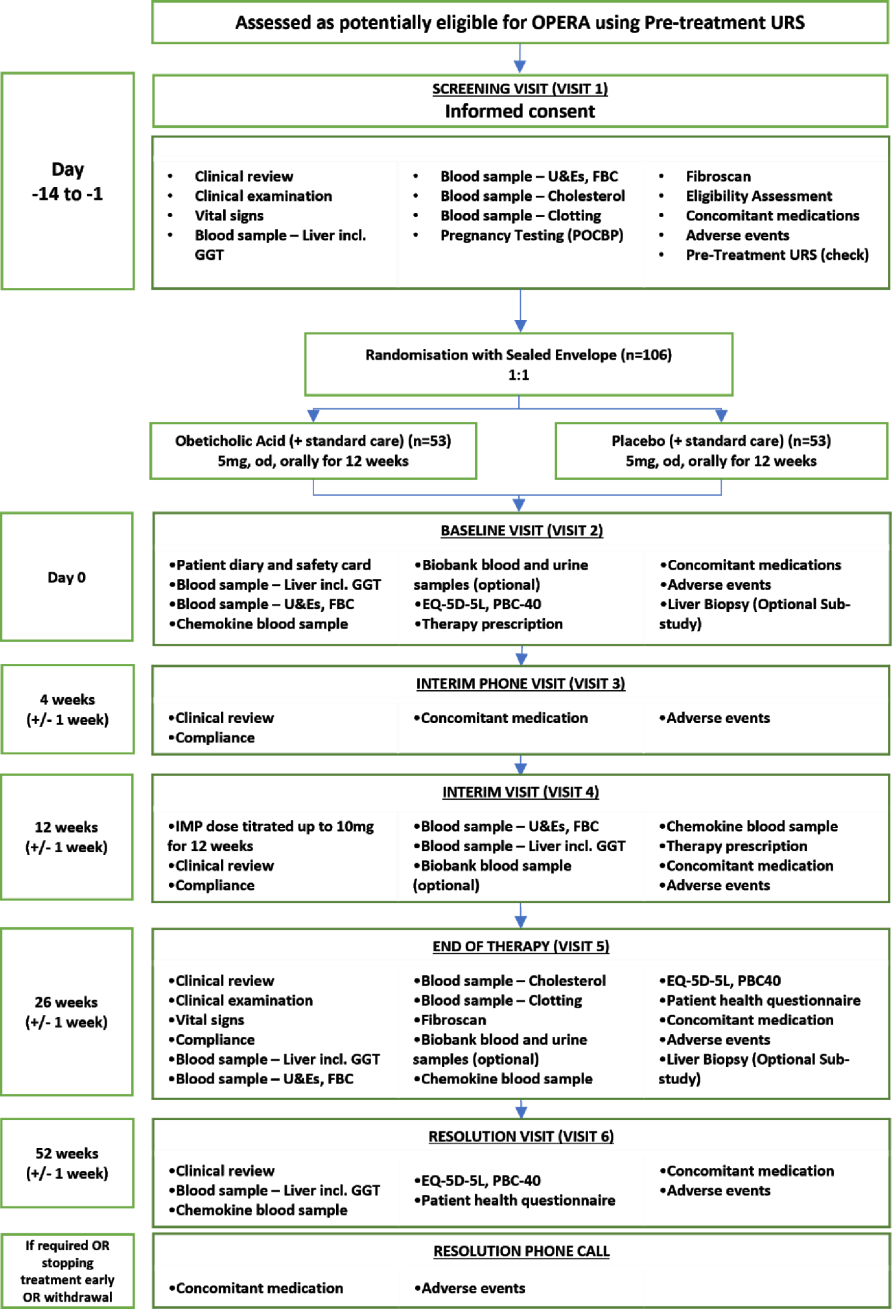
Participant flow diagram. FBC, full blood count; OPERA, Optimising Primary thErapy in pRimAry; PBC, primary biliary cholangitis; POCBP, people of childbearing potential; U&Es, urea and electrolytes; UDCA, ursodeoxycholic acid; URS, UDCA Response Score; GGT, gamma-glutamyl transferase; EQ-5D-5L, EuroQoL 5 Dimensions 5 Levels.

Participants will attend a total of up to five in-person visits and one phone call. These include the screening visit prior to randomisation and baseline visit, (screening and baseline visits may be concurrent if the patient has had recent useable blood results to use for screening), a visit at the end of the treatment phase and a final visit at the end of the follow-up phase. A detailed breakdown of the trial follow-up and assessments required at each visit for OPERA is illustrated in figure 1. The assessments to be conducted are as follows:

- Clinical review: Clinical history relevant to PBC and this trial, adapted according to the type of visit. At screening, this will include demographics, a detailed clinical review of PBC and its symptoms as well as medication history. At subsequent visits, this will include any changes in the history since screening, including any symptom changes or de novo symptom development and/or difficulties with taking medication.
- Clinical examination: A full physical examination will be conducted, including examination of skin; lymph nodes; eyes; ears; nose; throat; respiratory; cardiovascular; abdomen; musculoskeletal; neurological assessment; mental status assessment. Bodyweight will also be measured.
- Vital signs: Heart rate, blood pressure and temperature will be recorded.
- Blood sampling: A blood sample will be taken (maximum 50 mL) at times specified in figure 1 for the following assessments:
  - Renal function: urea, creatinine and serum electrolytes.
  - Liver biochemistry: alanine transaminase (ALT), aspartate aminotransferase (AST), alkaline phosphatase (ALP), total bilirubin, direct bilirubin, gamma-glutamyl transferase (GGT), albumin.
  - Cholesterol (total cholesterol, Low Density Lipoprotein (LDL), High Density Lipoprotein (HDL), total:HDL ratio and triglycerides).
  - Full blood count (haemoglobin, platelets and white cells—neutrophils and lymphocytes).
  - Clotting: Prothrombin time and activated partial thromboplastin time.
  - Experimental chemokine blood marker.
  - Optional biobank blood sample.
  Clinical samples will be analysed in the clinical laboratories of the trial site, following standard local laboratory procedures.
  All chemokine samples will be analysed simultaneously at the end of the trial. All chemokine assessments will be performed in a fully blinded manner, with regard to treatment group and pre-therapy and post-therapy timing.
  There are three optional additional blood samples (baseline, visits 4 and 5) for use in future PBC research.
- Urine sampling: Participants will be asked if they are willing to provide consent to give two optional urine samples (baseline and visit 5).
- URS: Predicts the likelihood of someone responding to UDCA.^10^ URS must be calculated using the OPERA URS calculator that is provided as part of this clinical trial and available here: https://www.mat.uniroma2.it/~alenardi/URS.html.
  The URS calculator considers three different ‘cut-offs’ to define UDCA response. In this trial, we use the version that considers the outcome of interest to be biochemical remission (ie, ALP <1× upper limit of normal (ULN)). Based on the URS value, a predicted probability of biochemical remission can be calculated. For the purposes of trial eligibility, URS will be calculated using blood results prior to treatment with UDCA.
- Pregnancy testing: For people of childbearing potential, a pregnancy test will be carried out during the trial screening visit.
- EQ-5D-5L: A five-item, validated general quality of life measure from which health utility can be calculated.^26^
- PBC-40: A disease-specific, patient-derived multidomain quality of life measure. It has six domains relating to fatigue, cognitive symptoms, social function, emotional status and general symptoms.^5^
- Patient Health Questionnaire: A non-validated 5-point scale to assess health status perceived by participants in response to the trial medication at 26 and 52 weeks.
- Transient elastography (fibroscan): Clinically validated non-invasive tool used to assess liver stiffness; a surrogate marker of liver fibrosis.
- Compliance: Compliance regarding IMP will be checked at the interim telephone visit (4 weeks after taking IMP at day 0) and followed up at a clinic visit at week 12 and week 26. IMP compliance will be recorded in the Clinical Data Management System (CDMS). Pharmacy will count and record all returns received from each participant on the IMP accountability log. An accountability check will also be performed as part of on-site monitoring visits.

### Optional liver biopsy sub-study

Liver biopsy pre and post therapy will be undertaken in a subgroup of participants (n=30) as part of an optional sub-study. Participants in the sub-study will be allocated in a ratio of 1:1 placebo:intervention via stratified randomisation. The procedure will be performed as for a normal clinical biopsy according to local centre practice. The blocks will be sectioned and stained for immuno-histochemistry analysis for senescence markers.

### Pharmacovigilance

All adverse events (AEs) occurring from consent to the end of trial participation will be recorded in the CDMS as well as the participant’s medical notes (with the exception of protocol specific reporting exclusions). AEs will be MedDRA coded; MedDRA, the Medical Dictionary for Regulatory Activities terminology, is the international medical terminology developed under the auspices of the International Council for Harmonisation of Technical Requirements for Pharmaceuticals for Human Use (ICH). MedDRA trademark is registered by ICH.

AEs meeting the seriousness criteria (serious AEs (SAEs)) will be reported within 24 hours of awareness. A SAE for the OPERA trial is any untoward medical occurrence that:

- Results in death
- Is life-threatening*
- Requires inpatient hospitalisation or prolongation of existing hospitalisation
- Results in persistent or significant disability/incapacity
- Consists of a congenital anomaly or birth defect
- Other important medical events that jeopardise the participant or require intervention to prevent one of the above consequences

*Life-threatening refers to an event in which the participant was at immediate risk of death at the time of the event; it does not refer to an event which hypothetically might have caused death if it were more severe. The assessment of expectedness will be performed by the CI against the approved Reference Safety Information (RSI) for the trial (SmPC for OCALIVA 5 mg, section 4.8 - Undesirable Effects). Suspected Unexpected Serious Adverse Reactions (SUSARs) will be reported to the MHRA (Medicines and Healthcare products Regulatory Agency) and REC (Research Ethics Committee) within the required regulatory reporting timelines.

In the event of a trial participant or their partner becoming pregnant during their participation in the trial, this will be reported within 24 hours of awareness.

Emergency unblinding is available via the 24-hour web-based randomisation system for valid medical or safety reasons.

### Discontinuation and withdrawal

Participants have the right to discontinue trial treatment or withdraw from the trial at any time without detriment to their care. Participants choosing to discontinue trial treatment will be invited to complete all trial follow-up visits and assessments. Participants choosing to withdraw from the trial will not take part in any further trial activity. Data collected up to the point of withdrawal will be retained and included in analysis, as per consent for the trial. At the point of withdrawal, participants can decide whether or not to allow the research team to continue to collect routine NHS data from their hospital records up to the 52-week visit time point. Participants who withdraw from the trial after randomisation will not be replaced.

NHS Indemnity applies in the OPERA trial and ensures compensation to those who suffer harm from trial participation but does not offer no-fault compensation.

### Data management and confidentiality

Participant data will be entered by site staff into a trial-specific CDMS, Sealed Envelope’s Red Pill (Sealed Envelope, London, UK). Access to the CDMS will be via individual, password-protected roles and limited to the user’s site and delegated duties. All potential participants will be assigned a unique Participant ID by site staff; the Participant ID will be used to add participants to the CDMS and will remain in place when participants are randomised into the trial. Personal data will be regarded as strictly confidential with all trial records and Investigator Site Files being stored at site with restricted access. Written consent will be obtained from participants to allow access to their hospital records.

Data will be handled, computerised, stored and archived in accordance with the General Data Protection Regulation (2018) and the latest Directive on Good Clinical Practice (GCP) (ICH-E6 (R3)). Newcastle Clinical Trials Unit (NCTU) staff will monitor the trial conduct and data integrity in accordance with the trial Monitoring Plan.

### Analysis

The primary analysis will be in the intention-to-treat (ITT) population and repeated in the per-protocol (PP) populations. There will be three analysis populations in total:

- ITT: Includes all participants who were randomised, analysed according to their allocated group. This extends to participants who are found to be ineligible post-randomisation and participants who, despite having withdrawn from treatment, have consented to continued follow-up.
- PP: Includes all participants who were randomised, received assigned treatment and achieved an 80% compliance rate to the intervention by week 26. Treatment compliance will be assessed using the tablet count of returned medication and calculated as the number of tablets taken divided by the number of tablets expected to have been taken.
- Safety population: Includes all participants who were randomised and received at least one dose of OCA or placebo.

All analyses will be described in a Statistical Analysis Plan, to be finalised prior to trial statisticians receiving any unblinded data. For the analysis of the primary outcome, we will use the augmented binary method.^25^ This will consist of fitting a bivariate normal regression model to the alkaline phosphatase (ALP) and bilirubin values (expressed as a percentage of the ULN). The model will include parameters representing the treatment effect and the effect of baseline measurements of the respective component. Model parameters will then be used to estimate the OR of the normalisation rate between arms. A 95% CI and p value for testing the null hypothesis of no difference between arms will be obtained via the delta method and Wald test, respectively. As a secondary analysis, we will also analyse the normalisation rate as a binary outcome using a logistic regression with the same parameters. Continuous secondary outcomes will be analysed with linear mixed effects models, adjusting for baseline. Binary outcomes will be analysed with a logistic regression model (and, where the outcome is defined by a dichotomised continuous variable, with the augmented binary method). Should there be a non-negligible level of missing data in the primary outcome (>5% loss to follow-up), we will consider performing a sensitivity analysis using multiple imputation techniques. The linear mixed effects model accounts for missing outcome data under a missing at random assumption.

### Sample size calculations

The proposed sample size is 106 (powered at 48 per group for two groups with a 10% increase for drop out). This sample size has been derived based on an analysis of the extensive UK-PBC dataset undertaken as part of the design of this trial, as well as published Dutch and Global PBC analyses.^12 27 28^ Based on the patients who would fulfil the eligibility requirements, the normalisation rate at 12 months post-UDCA was 17%. To account for the placebo effect, we assume the 6 month control arm normalisation rate will be 20%. We power to detect a significant difference when the experimental arm has a 45% normalisation rate. If the normalisation rate were analysed as binary, then 144 patients would be required to attain 90% power (5% two-sided error) with a two-arm design, not allowing for dropout (Pearson χ^2^ test). As the normalisation rate is defined in terms of ALP and bilirubin values (continuous variables) being below an upper limit, the augmented binary method^25^ can be used. This method makes inference on the normalisation rate but uses the continuous data on ALP and bilirubin to improve the precision. Simulation studies that resampled data from the UK-PBC data to simulate trials under the null and alternative hypotheses were used to determine that with the augmented binary analysis, the power is 90% when the number of patients per arm is 48 (which we increase by 10% to 53 to account for 10% dropout). Thus, the sample size required is 106.

### Trial management and oversight

The trial is sponsored by The Newcastle on Tyne Hospitals NHS Foundation Trust and has independent oversight from a Data Monitoring Committee (DMC) and a Trial Steering Committee (TSC).

The DMC and TSC consist of independent experts in the field and patient and public representatives. Each committee has a charter, agreed by the independent members, defining their roles and responsibilities. The trial will be co-ordinated by a Trial Management Group (TMG) that will include co-applicants and individuals responsible for the day-to-day management of the trial. The NCTU will be responsible for communicating protocol amendments to participating sites and carrying out central, remote, off-site and on-site monitoring.

### Patient and public involvement

The trial team have, for over 20 years, worked closely with the PBC Foundation (a national PBC patient group with over 10 000 PBC patient members, https://www.pbcfoundation.international/) and LIVErNORTH (a regional liver patient support group with over 2000 patients http://www.livernorth.org.uk/). among the PBC patient community, effective treatment of symptoms is seen as a key research priority. Patient representatives from these two groups have been actively involved in priority setting which led to the trial, and in the development of both the concept, trial design and development of trial documentation. Independent PPI members have continued oversight of the trial and will input on results dissemination.

## Ethics and dissemination

Favourable ethical opinion was received from London – Riverside Research Ethics Committee (reference: 22/LO/0878). Potential participants will be fully informed of their rights and the benefits and harms of the trial by the research team before giving informed consent to participate in the trial (see online supplemental material for consent form). Results will be disseminated in peer-reviewed publications, at national and international conferences, in peer-reviewed journals and to participants and the public (using lay language).

## Discussion

We believe that this trial is important in the field of PBC and has five particular strengths. The first is that it explores a new paradigm for disease-modifying treatment of PBC; risk-informed early treatment stratification with patients at increased risk offered an enhanced, combination approach to therapy. This potentially avoids the delay to optimal treatment that is inherent in the current ‘step-up’ treatment model, where the highest risk patients will wait for at least a year for effective treatment. We propose that the delay in optimal treatment is important in PBC; there is now evidence to show that a critical disease process, ductopenia with physical loss of the small intra-hepatic bile ducts, is already present at diagnosis in the highest risk patients.^29^ Given that this progressive process is only controlled, let alone reversed, by the more potent second-line therapies, to delay those therapies for arbitrary process reasons is completely counterintuitive. We also believe that the current step-up model hinders effective therapy as there is now comprehensive data from the UK to show that the movement to addition of second-line therapy occurs in only around half of eligible patients.^30^ Poor clinician awareness and understanding of the inherently complex, multi-stage step-up approach, which requires several decision points and multiple follow-up appointments, is a major contributing factor.^31^ We believe a simpler treatment model, with definitive treatment started at the outset of the disease journey (in patients who would benefit from it), would significantly improve treatment ‘reach’.

The second strength is that it will, for the first time, explore the degree to which the cardinal disease process thought to underpin ductopenia, that of bile duct cell senescence,^29^ is directly reversed by OCA. No previous trials of this agent in PBC have addressed molecular mechanisms of action, focusing as they have on surrogate biochemical markers of action (which created significant issues around drug licensing).^30^ This question is highly pertinent due to two factors; the first is that preclinical modelling in both mouse models of cholestasis and in human cell culture lines, both of which provide further evidence to suggest a key role for senescence in bile duct cell injury, suggests that OCA does indeed reverse cellular senescence.^32^ The second, linked factor is that in contrast to OCA, peroxisome proliferator-activated receptor agonists (another class of agent widely used for second-line therapy in PBC) were in the same models, not shown to be anti-senescent^29^ (instead, the actions are mainly on the biochemical aspects of cholestasis), potentially a key issue. Decisions as to which therapy to use in PBC are currently made around improvement in blood biochemistry tests, disease stage and presence of pruritus. Definitive treatment for PBC will need us to take a much more nuanced view of the actions of key drugs. By exploring the actions of OCA (and placebo) on senescence, this trial will provide important mechanistic evidence which will help us further refine therapy in PBC.

The third strength is that it uses, for the first time, baseline stratification of risk to guide therapy. The models used to date have been predicated on stratification ‘after the fact’ (ie, where actual response was used to determine whether additional therapy is needed). This leads, we believe, to the issues outlined above. It is clear, however, that the likelihood of response to UDCA can be predicted with accuracy at the outset of disease, and algorithms developed to allow prediction in practice.^10^ The application of models such as this to stratify at disease baseline, in the way we are doing in this trial, has been advocated in the field.^33^

The fourth strength is that this trial demonstrates how the use of innovative statistical methods can help improve efficiency for rare diseases. This trial represents the first time that the augmented binary method^25^ has been used prospectively to set the power of the trial at 90% while reducing the sample size required for this level of power. The reduction of sample size by more than 30% led to a substantial reduction in the cost and length of the trial and will hopefully highlight the method for use in other trials using similar ‘response’ endpoints.

The final strength is that it uses normalisation of liver biochemistry as the surrogate trial end-point, rather than reduction to below thresholds of abnormality which has been the approach to date. This change in treatment target has been advocated by expert groups and is based on two forms of evidence, both of which suggest residual disease activity in patients meeting conventional UDCA response criteria but in whom liver blood tests are abnormal. First there is clinical survival data to suggest that normalisation is associated with the lowest risk to long-term survival.^34^ Second, mechanistic studies have suggested ongoing inflammatory activity in this group of persistently abnormal biochemistry ‘responders’.^16^

The key weakness of the OPERA trial is that it, as has been the case with all trials of novel therapy in recent years, utilises a surrogate marker (blood liver biochemistry) as the primary end-point. Although the markers used, ALP and bilirubin are highly predictive of hard outcomes such as death or liver transplant and are used routinely in clinical practice on this basis, they remain surrogate markers. The issue of surrogate markers, their use in PBC and their acceptability to regulators has been a topic of significant recent controversy.^35^ Placebo-controlled trials of long-term therapy in a chronic disease such as PBC are very challenging indeed, especially when second-line therapies are available on a conditional approval basis following failure of UDCA therapy. Although OPERA still relies on surrogate markers, two of the additional endpoints used, liver biopsy changes in response to therapy and the degree to which early therapy changes the trajectory of the disease, are closer to the biological basis of the disease and thus, arguably, do represent an advance of the conventional markers. A second limitation of the trial is that it explores the actions of only one of the advanced PBC therapies. The advent of alternative second-line therapies (elafibranor and seladelpar) as licensed treatments occurred well after the design and commencement of OPERA. Were the trial to be designed today, it would be highly desirable to explore each of the agents for primary therapy use utilising an advanced trial methodology.

PBC as a disease has seen significant progress in the last few years, with new drugs being licensed. We believe, however, that we are now at the point where the main limitation is not the drugs but how we choose to use them and, in particular, a lack of evidence to support such choices. We believe that this trial approach addresses all the limitations of the current therapy model in a single protocol. We would welcome its use in the evaluation of other potent PBC therapies.

### Trial status

This manuscript is based on trial protocol V.6.0 dated 27 October 2025. The first patient was recruited in January 2024.

## Supplementary material

10.1136/bmjopen-2025-113812 online supplemental file 1

## References

[1] Griffiths L, Dyson JK, Jones DEJ (2014). The new epidemiology of primary biliary cirrhosis. Semin Liver Dis.

[2] Carbone M, Mells GF, Pells G (2013). Sex and age are determinants of the clinical phenotype of primary biliary cirrhosis and response to ursodeoxycholic acid. Gastroenterology.

[3] Dyson JK, Wilkinson N, Jopson L (2016). The inter-relationship of symptom severity and quality of life in 2055 patients with primary biliary cholangitis. Aliment Pharmacol Ther.

[4] Goldblatt J, Taylor PJS, Lipman T (2002). The true impact of fatigue in primary biliary cirrhosis: a population study. Gastroenterology.

[5] Jacoby A, Rannard A, Buck D (2005). Development, validation, and evaluation of the PBC-40, a disease specific health related quality of life measure for primary biliary cirrhosis. Gut.

[6] Mells GF, Pells G, Newton JL (2013). Impact of primary biliary cirrhosis on perceived quality of life: the UK-PBC national study. Hepatology.

[7] Newton JL, Hollingsworth KG, Taylor R (2008). Cognitive impairment in primary biliary cirrhosis: symptom impact and potential etiology. Hepatology.

[8] Newton JL, Pairman J, Sutcliffe K (2008). A predictive model for fatigue and its etiologic associations in primary biliary cirrhosis. Clin Gastroenterol Hepatol.

[9] Rice S, Albani V, Minos D (2021). Effects of Primary Biliary Cholangitis on Quality of Life and Health Care Costs in the United Kingdom. Clin Gastroenterol Hepatol.

[10] Carbone M, Nardi A, Flack S (2018). Pretreatment prediction of response to ursodeoxycholic acid in primary biliary cholangitis: development and validation of the UDCA Response Score. Lancet Gastroenterol Hepatol.

[11] Harms MH, de Veer RC, Lammers WJ (2020). Number needed to treat with ursodeoxycholic acid therapy to prevent liver transplantation or death in primary biliary cholangitis. Gut.

[12] Lammers WJ, van Buuren HR, Hirschfield GM (2014). Levels of alkaline phosphatase and bilirubin are surrogate end points of outcomes of patients with primary biliary cirrhosis: an international follow-up study. Gastroenterology.

[13] Trivedi PJ, Lammers WJ, van Buuren HR (2016). Stratification of hepatocellular carcinoma risk in primary biliary cirrhosis: a multicentre international study. Gut.

[14] Trivedi PJ, Hirschfield GM (2015). Primary biliary cirrhosis: Renaming primary biliary cirrhosis-clarity or confusion?. Nat Rev Gastroenterol Hepatol.

[15] Younossi ZM, Stepanova M, Golabi P (2019). Factors Associated With Potential Progressive Course of Primary Biliary Cholangitis: Data From Real-world US Database. J Clin Gastroenterol.

[16] Jones DEJ, Wetten A, Barron-Millar B (2022). The relationship between disease activity and UDCA response criteria in primary biliary cholangitis: A cohort study. EBioMedicine.

[17] Liu JZ, Almarri MA, Gaffney DJ (2012). Dense fine-mapping study identifies new susceptibility loci for primary biliary cirrhosis. Nat Genet.

[18] Yagi M, Matsumoto K, Komori A (2020). A validation study of the Ursodeoxycholic Acid Response Score in Japanese patients with primary biliary cholangitis. Liver Int.

[19] (2017). Obeticholic acid for treating primary biliary cholangitis (2017) Technology appraisal guidance TA443. https://www.nice.org.uk/guidance/ta443.

[20] Nevens F, Andreone P, Mazzella G (2016). A Placebo-Controlled Trial of Obeticholic Acid in Primary Biliary Cholangitis. N Engl J Med.

[21] Murillo Perez CF, Ioannou S, Hassanally I (2023). Optimizing therapy in primary biliary cholangitis: Alkaline phosphatase at six months identifies one-year non-responders and predicts survival. Liver Int.

[22] Zhang L-N, Shi T-Y, Shi X-H (2013). Early biochemical response to ursodeoxycholic acid and long-term prognosis of primary biliary cirrhosis: results of a 14-year cohort study. Hepatology.

[23] Abbas N, Culver EL, Thorburn D (2023). UK-Wide Multicenter Evaluation of Second-line Therapies in Primary Biliary Cholangitis. Clin Gastroenterol Hepatol.

[24] Barron-Millar B, Ogle L, Mells G (2021). The Serum Proteome and Ursodeoxycholic Acid Response in Primary Biliary Cholangitis. Hepatology.

[25] Wason J, McMenamin M, Dodd S (2020). Analysis of responder-based endpoints: improving power through utilising continuous components. Trials.

[26] Herdman M, Gudex C, Lloyd A (2011). Development and preliminary testing of the new five-level version of EQ-5D (EQ-5D-5L). Qual Life Res.

[27] Kuiper EMM, Hansen BE, Lesterhuis W (2011). The long-term effect of ursodeoxycholic acid on laboratory liver parameters in biochemically non-advanced primary biliary cirrhosis. Clin Res Hepatol Gastroenterol.

[28] Murillo Perez CF, Hirschfield GM, Corpechot C (2019). Fibrosis stage is an independent predictor of outcome in primary biliary cholangitis despite biochemical treatment response. Aliment Pharmacol Ther.

[29] Hardie C, Green K, Jopson L (2016). Early Molecular Stratification of High-risk Primary Biliary Cholangitis. EBioMedicine.

[30] Abbas N, Smith R, Flack S (2024). Critical shortfalls in the management of PBC: Results of a UK-wide, population-based evaluation of care delivery. *JHEP Rep*.

[31] Jopson L, Khanna A, Peterson P (2018). Are Clinicians Ready for Safe Use of Stratified Therapy in Primary Biliary Cholangitis (PBC)? A Study of Educational Awareness. Dig Dis Sci.

[32] Gee LMV, Barron-Millar B, Leslie J (2023). Anti-Cholestatic Therapy with Obeticholic Acid Improves Short-Term Memory in Bile Duct-Ligated Mice. Am J Pathol.

[33] Marschall HU (2018). Ensuring timely treatment of patients with primary biliary cholangitis. Lancet Gastroenterol Hepatol.

[34] Corpechot C, Lemoinne S, Soret P-A (2024). Adequate versus deep response to ursodeoxycholic acid in primary biliary cholangitis: To what extent and under what conditions is normal alkaline phosphatase level associated with complication-free survival gain?. Hepatology.

[35] Jones DEJ, Beuers U, Bonder A (2024). Primary biliary cholangitis drug evaluation and regulatory approval: Where do we go from here?. Hepatology.

