# Supplementary material for: Optimising Primary thErapy in pRimAry biliary cholangitis (OPERA): protocol for a randomised, double-blind, placebo-controlled trial of enhanced primary therapy with obeticholic acid
Source: BMJ Open. 2026 Mar 11;16(3):e113812. doi: 10.1136/bmjopen-2025-113812 (PMC12983740; doi:10.1136/bmjopen-2025-113812)
Supplement: online supplemental file 1 [file bmjopen-16-3-s001.pdf]

Local NHS trust logo

Optimising Primary Therapy in Primary Biliary Cholangitis

INFORMED CONSENT FORM

Version 3.0 04th July 2024

CONFIDENTIAL ONCE COMPLETED

Participant ID: | | | | | | |

Name of Principal Investigator: ___________________________________

Please INITIAL the boxes if you agree:

1.	I confirm that I have read the Participant Information Sheet dated _ _ /_ _ _ /_ _ _ _ version ___ for the above study and that I have had the opportunity to consider the information, ask questions and have had these answered satisfactorily.	
2.	I understand that I do not have to take part in this study and that I am free to withdraw at any time without giving a reason, without my medical care or legal rights being affected.	
3.	I understand that if I withdraw from the study, the information and samples collected from me until that point will be kept and used.	
4.	I understand that if I choose to stop taking part in the study, the researchers will continue collecting information about my health from my hospital records for the duration of the study, unless I ask for this to stop.	
5.	I give permission for parts of my medical records and the information collected during the study to be accessed by people responsible for the conduct of the study, including those from Newcastle University, the Newcastle upon Tyne Hospitals NHS Foundation Trust and national regulatory authorities.	
6.	I understand that the information provided in this study is being managed by the Newcastle Clinical Trials Unit, part of Newcastle University.	
7.	I understand that the information held and maintained by <Site to localise> about me will be used by the study team at the hospital to contact me or provide information about my health status.	
8.	I understand that the information collected from me for the study will be stored on a study database provided by Sealed Envelope™. This will be kept confidential and will be stored securely. I will only be identified on this database through a unique study number and date of birth, sex and ethnicity.	

9.	I understand that any personal information collected about me for the study will be kept confidential and will not be made public. I understand that data from the study, will be published in medical journals, at research meetings and shared with other researchers. I understand that data from the study will be de-identified and that I will not be directly identified in published results.	
10.	I understand that the information collected about me will be used to support other research in the future and may be shared anonymously with other researchers.	
11.	I understand that my GP and hospital care team will be informed of my participation in this study. I understand there may be necessary exchange of information about me between the study team at <Site to localise> , my GP and hospital doctor, including the results of medical tests.	
12.	I agree to the information provided and this signed consent form being stored for 5 years after the end of the study.	
13.	I give my permission for a copy of this consent form to be sent securely to the Newcastle Clinical Trials Unit for review in accordance with Good Clinical Practice.	
14.	**I agree to take part in the OPERA study.**	

**For participants who have the potential to become pregnant only:**		
15.	I understand that I will have to use at least an acceptable effective form of contraception as outlined in the Participant Information Sheet, if sexually active.	
16.	I understand that I will need to provide a urine sample to ensure that I am not pregnant. I understand that this is for safety reasons.	
17.	I understand that if I become pregnant during the trial the research team will follow my pregnancy to completion and for up to one year after birth. This may include parts of my child's medical records being looked at by people responsible for the conduct of the study.	

**For male participants only:**		
18.	I understand that if my partner becomes pregnant during the study, they will be asked to provide consent to allow the research team to follow their pregnancy to completion and for up to one year after birth.	

**OPTIONAL for ALL participants:**		
19.	I consent to my remaining blood samples and sample information being stored at Newcastle Biobank and being used in future PBC research (including research outside of the UK).	

20.	I consent to provide three optional blood samples (approx. 1 teaspoon each) as part of this research trial, and for these samples and sample information being stored at Newcastle Biobank and being used in future PBC research (including research outside of the UK).	
21.	I understand that as part of the trial some of the blood samples collected from me will be analysed by researchers at Newcastle University. The researchers will not know my name, and I will be identified on these samples through a unique study number.	
22.	I consent to provide two optional urine samples as part of this research trial, and for these samples and linked data being stored at Newcastle Biobank and being used in future PBC research (including research outside of the UK).	
23.	I would like a summary of the results to be sent to me when the study has finished.	

**OPTIONAL BIOPSY SUB-STUDY participants:**		
24.	I would like to take part in the OPERA biopsy sub-study. I understand that this involves up to two liver biopsies; one at the start of the trial, unless I have already had a biopsy in the last 6 months, and one during the trial.	
25.	I understand that as part of the sub-study the biopsy samples collected from me will be analysed for the trial by researchers at NovoPath (based at the Newcastle upon Tyne Hospitals NHS Foundation Trust). The researchers will not know my name, and I will be identified on these samples through a unique study number.	
26.	If I have had a liver biopsy within the last 6 months, I agree that this tissue can be used for the OPERA trial and understand that I will not require a biopsy at the start of the trial.	

STOP and check that you have initialled relevant boxes

Name of Participant	Date	Signature
Name of Person Receiving Consent	Date	Signature

When completed: File original in the investigator site file; Provide 1 copy for the study participant; File/upload 1 copy to the participant's medical notes.

Send a copy of this form by secure email (e.g. nhs.net) to nctu.opera.conf@nhs.net
